# Cellular and molecular signaling towards T cell immunological self-tolerance

**DOI:** 10.1016/j.jbc.2024.107134

**Published:** 2024-03-02

**Authors:** Fortunata Carbone, Claudia Russo, Alessandra Colamatteo, Claudia La Rocca, Clorinda Fusco, Alessandro Matarese, Claudio Procaccini, Giuseppe Matarese

**Affiliations:** 1 Laboratorio di Immunologia, Istituto per l'Endocrinologia e l'Oncologia Sperimentale “G. Salvatore”, Consiglio Nazionale delle Ricerche (IEOS-CNR), Napoli, Italy; 2 Unità di Neuroimmunologia, IRCCS-Fondazione Santa Lucia, Roma, Italy; 3 D.A.I. Medicina di Laboratorio e Trasfusionale, Azienda Ospedaliera Universitaria “Federico II”, Napoli, Italy; 4 Treg Cell Lab, Dipartimento di Medicina Molecolare e Biotecnologie Mediche, Università degli Studi di Napoli “Federico II”, Napoli, Italy; 5 Dipartimento di Medicina Clinica e Chirurgia, Università degli Studi di Napoli “Federico II”, Naples, Italy

**Keywords:** TCR, regulatory T cells, immunological *self*-tolerance, CTLA-4, IL-2, CD45

## Abstract

The binding of a cognate antigen to T cell receptor (TCR) complex triggers a series of intracellular events controlling T cell activation, proliferation, and differentiation. Upon TCR engagement, different negative regulatory feedback mechanisms are rapidly activated to counterbalance T cell activation, thus preventing excessive signal propagation and promoting the induction of immunological self-tolerance. Both positive and negative regulatory processes are tightly controlled to ensure the effective elimination of foreign antigens while limiting surrounding tissue damage and autoimmunity. In this context, signals deriving from co-stimulatory molecules (*i.e.*, CD80, CD86), co-inhibitory receptors (PD-1, CTLA-4), the tyrosine phosphatase CD45 and cytokines such as IL-2 synergize with TCR-derived signals to guide T cell fate and differentiation. The balance of these mechanisms is also crucial for the generation of CD4^+^ Foxp3^+^ regulatory T cells, a cellular subset involved in the control of immunological self-tolerance. This review provides an overview of the most relevant pathways induced by TCR activation combined with those derived from co-stimulatory and co-inhibitory molecules implicated in the cell-intrinsic modulation of T cell activation. In addition to the latter, we dissected mechanisms responsible for T cell–mediated suppression of immune cell activation through regulatory T cell generation, homeostasis, and effector functions. We also discuss how imbalanced signaling derived from TCR and accessory molecules can contribute to autoimmune disease pathogenesis.

One of the most important functions of the immune system is to mount a specific and efficient response against foreign antigens, but at the same time, since the uncontrolled immune cell activation could be dangerous to the host, this process must be finely tuned to limit and/or prevent excessive damage to surrounding tissues. In addition, immune cells need to maintain a state of immunological tolerance towards self-antigens to avoid the development of autoimmune diseases, suggesting the existence of a number of regulatory mechanisms controlling immune cell activation and response. The nature and outcome of T cell response are influenced by multiple pathways, including T cell receptor (TCR)-mediated signaling, which plays a central role in controlling T cell function and drives the immune response towards either effector or tolerogenic commitment. In this context, both the strength and duration of the TCR-mediated signals influence T cell activation and fate decision. TCR pathway engagement is triggered by the recognition of a specific peptide presented in the context of either class I or class II major histocompatibility complex (MHC) molecules expressed on the surface of antigen presenting cells (APCs) (first signal); together with this signal, co-stimulatory and co-inhibitory molecules expressed on the T cell surface transduce signals into the cells (second signal) that synergize with TCR signaling and positively or negatively modulate its pathway, leading to either activation, proliferation, and cytokine secretion or to anergy and apoptotic death, respectively (1). Interestingly, the expression of these co-inhibitory molecules is often induced upon T cell engagement in the presence of co-stimulation, suggesting the presence of a negative feedback loop controlling the immune system activation. The key role of these coinhibitory molecules in the control of immune cell activation is confirmed by the observation that their genetic ablation leads to the development of several autoimmune diseases, mainly associated with overactive T cell response (2). Activation of TCR signaling cascade is associated with a series of phosphorylation events triggered by the LCK–ZAP-70 axis. These events lead to the activation of downstream enzymes, including PLC-γ1, PI3K, and MAPK, with consequent propagation of the signal (3). The succession of phosphorylation and de-phosphorylation reactions is the most flexible and immediate tool for regulating the TCR-signaling cascade, although protein modifications, such as ubiquitylation and deubiquitylation, can also contribute to the net signaling output (4). In addition to the aforementioned mechanisms of TCR regulation, the affinity between the TCR and the MHC:peptide complex, their quantitative expression on the T cell surface, the timing and qualitative nature of their interaction can also control T cell function and determine their fate (5).

The regulatory negative mechanisms, that act rapidly and are generated in parallel with TCR-mediated activating signals, have been extensively characterized and can modulate T cell activation, ensuring the generation of appropriate responses to external stimuli. In this context, a recent paper by Chan *et al.* has shown that programmed cell death protein 1 (PD-1) plays a key role in TCR-induced signaling inhibition. Specifically, it has been reported that PD-1–mediated inhibition was able to exclusively target pathways activated by TCR engagement, independently of CD28 contribution, and it was highly sensitive to both the intrinsic quality of the ligand and the density of peptide:MHC complex on the surface of APCs (6).

It has now become clear that signals from the TCR and from co-stimulatory and co-inhibitory molecules are essential not only for limiting and directing T cell activation but also for controlling the development and suppressive function of CD4^+^ regulatory T (Treg) cells. This T cell–specialized subset expresses the transcription factor Foxp3 and is involved in the negative regulation of immune-mediated inflammation and in the maintenance of immune tolerance towards self-antigens (7). Several studies have revealed that also in the thymus, the strength and the affinity of interactions between TCR and MHC-bound self-peptides play a key role in driving T cell fate towards clonal deletion *versus* either maturation into conventional T (Tconv) cells or differentiation into thymic-derived Treg (tTreg) cells, also called “natural” Treg cells. Moreover, in the periphery, TCR-mediated signaling is also able to regulate the conversion of naive CD4^+^ T cells into the so-called “peripheral” Treg (pTreg) cells. However, other signals are also involved in the balance of these processes, as multiple T cell–associated factors can influence the overall strength of TCR signaling.

In this review, we will mainly focus on the most recent findings concerning the integration and coordination of distinct signals derived from the TCR, the co-stimulatory and co-inhibitory molecules (*e.g.*, CD80, CD86, PD-1, B7-1, CTLA-4), the tyrosine phosphatase CD45 and cytokines (such as IL-2), with particular emphasis on their role in controlling the functional outcome of T cell responses and the homeostasis of Treg cells.

## Role of TCR signaling in the control of immunological self-tolerance

TCR signaling is controlled by several mechanisms that differ in time of action and/or target molecules. The biological outcome of TCR binding is influenced by several parameters, including the avidity of the ligand, the duration of binding, and the presence and type of accessory molecules on APCs. In this context, several membrane-bound scaffold proteins and cytoplasmic adaptor molecules have been shown to turn off TCR signaling in T cells by recruiting protein and lipid kinases and/or phosphatases. Among the phosphatases, PTPN22 regulates TCR signaling by binding the c-SRC kinase and inhibiting LCK activity in effector and memory T cells (8). Consistent with this evidence, mutations altering the PTPN22–c-SRC kinase interaction are associated with increased phosphatase activity, a condition that increases the risk of autoimmune diseases (9). Similarly, in the absence of the cytoplasmic protein tyrosine phosphatase SHP1, there is increased positive and negative thymocyte selection and T-cell activation resulting in the development of autoimmunity (10). Another mechanism of regulation of TCR activation is related to the half-life of the MHC:peptide complex interaction with the TCR (11) and indeed, mutations in the peptide (reducing this interaction) are associated with a disproportionate reduction in TCR-mediated signaling (12). This mechanism prevents TCR activation induced by its interaction with weak ligands and conversely allows signal transmission only in response to receptor–ligand interactions above a certain strength and threshold (13).

It is important to emphasize that the biological outcome of T cell response is also related to the intensity of the signal deriving from TCR stimulation by the peptide:MHC complex, which can generate an oscillatory, sustained, or transient response. In particular, oscillatory responses, which are for example induced by weak ligands in double-positive (DP) thymocytes, promote cell survival (14), whereas strong stimuli induced by agonist peptide:MHC complexes can lead to DP thymocyte death (15). On the other hand, lower amplitude but longer lasting signals produced by weaker stimuli, such as those deriving from antagonist or weak agonist peptide:MHC complexes, can induce DP thymocyte differentiation (15). In addition, suboptimal stimuli can induce T cell anergy or Treg cell generation (16). In summary, gene expression profiles and defined biological outcomes are likely to be determined by the amplitude and timing of TCR-mediated signals.

### Role of TCR signaling in thymic Treg cell generation

Several lines of research have supported the key role of TCR-mediated signaling in the control of T cell fate and their differentiation towards effector or regulatory compartment. Specifically, both TCR-ligand affinity and signal strength have been shown to regulate the generation and function of both tTreg and pTreg cells. At the central level, the affinity of TCR for self-peptide is fundamental in controlling positive selection of CD4^+^CD25^+^ thymocytes; indeed, TCRs with low peptide affinity preclude CD25^+^ development, while higher TCR affinity for self-peptide promotes the thymic selection of CD4^+^CD25^+^ Treg cells (17). The analysis of a panel of TCRs with a broad range of reactivity toward a given antigen revealed that a TCR with higher affinity was associated with an increased absolute number of tTreg cells (18). As concerns the role of TCR-dependent signal strength, tTreg cell precursors are induced by stronger TCR signals than Tconv cells (19); in addition, it has been reported that the generation of Foxp3^+^ tTreg cells can be sustained only by the binding of a self-peptide agonist to a strong reactive TCR. On the contrary, the use of a partial self-peptide agonist resulted in thymocyte deletion with failure in Treg cell generation, thus suggesting that TCR-mediated signal quality is able to guide the fate of autoreactive thymocytes toward their elimination or differentiation into Treg cells (20). TCR affinity is a critical factor also for tissue-specific Treg cell function during autoimmunity; indeed, it has been observed that high expression of Treg-associated markers, such as T-bet, GITR, CTLA-4, and ICOS, was strongly correlated with TCR signal strength (21). However, although Treg cells are characterized by a more autoreactive TCR as compared to Tconv cells, both high- and low-affinity Treg cells contributed to protection from autoimmune diabetes, albeit through different mechanisms. Specifically, it was observed that Treg cells with a high-affinity TCR were characterized by increased expression of IL-10, TIGIT, GITR, and CTLA-4, while those with low affinity showed increased transcripts for *Areg* and *Ebi3*. These data suggested that the degree of TCR affinity for self-antigen may influence the mechanisms by which autoimmunity is controlled (22).

Although the precise signaling pathways governing TCR-mediated Treg commitment still remain enigmatic, recent findings have elucidated some of the mechanisms by which different signaling molecules can modulate the strength of the TCR signal, thus controlling Treg cell generation. In this context, O'Hagan *et al.* have demonstrated that the actin cytoskeletal remodeling protein Pak2 played a key role in tTreg cell development by controlling the strength of the TCR signal; indeed, Pak2−/− thymocytes exhibited reduced TCR signaling, and mice with T cell–specific deletion of this molecule were characterized by reduced number of tTreg cells and developed spontaneous autoimmune colitis. Furthermore, Pak2 deficiency in thymocytes has been associated with impaired STAT5 activation following IL-2 stimulation, suggesting that, in addition to the generation of strong TCR signals, Pak2 also regulated tTreg cell development by controlling IL2-mediated signals (23).

**Figure 1:**
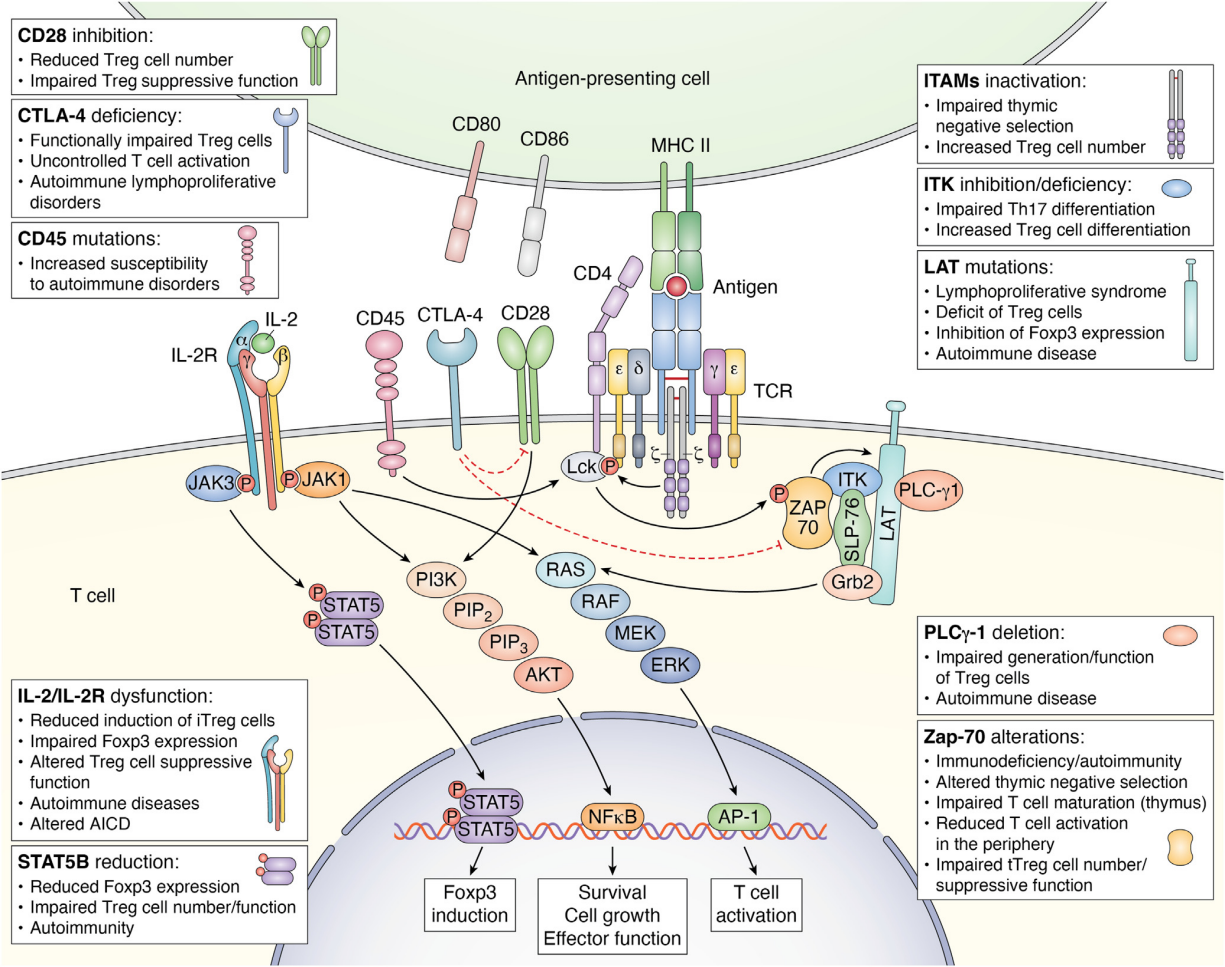
**Schematic representation of the main alterations in TCR signaling pathway and accessory molecules associated with autoimmunity/immunodeficiency.** The major signaling pathways induced by TCR engagement, combined with those derived from CD28 costimulatory molecule, CTLA-4 inhibitory receptor, the tyrosine phosphatase CD45 and IL-2R, involved in the control of T cell activation, and establishment of immunological *self*-tolerance. In the *gray* boxes are listed the alterations/pathological conditions associated with dysfunctions of the reported molecules. TCR, T cell receptor.

A growing body of evidence has revealed that perturbation of TCR downstream pathways and dysregulation of their key enzymes are associated with pathological conditions, such as immunodeficiency and autoimmunity (Fig. 1). Specifically, loss of ZAP-70 results in severe immunodeficiency associated with impaired T cell maturation in the thymus and their reduced activation in the periphery (24); at the same time, single-nucleotide substitution of ZAP-70 is associated with autoimmunity. Indeed, mice expressing a phosphorylation-defective mutant of ZAP-70, with consequent attenuation of TCR signaling, spontaneously developed autoimmune arthritis and were characterized by an altered negative selection of self-reactive T cells, together with a reduced number of tTreg cells with an impaired suppressive function (25, 26). In line with these studies, mice with ZAP-70 mutations resulting in either reduction of its levels or its inability to recruit TCR downstream effector molecules were characterized by impaired tTreg cell generation and altered deletion of autoreactive T cells with no impact on thymic differentiation of CD4^+^ and CD8^+^ T cells (27, 28) (Fig. 1). Several ZAP-70 amino acid substitutions, leading to a stepwise reduction in TCR-mediated signaling, can lead to autoimmune or immunocompromised conditions, depending on the reached signaling threshold, which is responsible for the opposing effects of TCR-mediated pathways on thymic selection and immunological self-tolerance establishment. Specifically, a ZAP-70 variant that can only moderately reduce TCR signaling has been shown to exert no effect on thymic selection, while a more severe ZAP-70 defect abolished thymic positive selection, leading to immunodeficiency. Crossing these two mutations had a more consistent effect on the process of thymic negative selection and the generation of tTreg cell, whose impairment resulted in excessive autoantibody and hyper-Immunoglobulin E production (29). Alterations in ZAP-70 function that dampen TCR signaling can be at the basis of several autoimmune diseases by altering positive selection of those thymocytes that should be physiologically eliminated. However, attenuation of the TCR signal associated with ZAP-70 alterations has a greater impact on the Treg cell compartment than Tconv cells, since Foxp3 physiologically represses ZAP-70 expression, further inhibiting TCR signaling; as a result, reduction of TCR signaling intensity generates self-reactive Tconv cells and simultaneously impairs the development and function of Treg cells (30). Recent studies have shown that the activity of ZAP-70 as a scaffold protein, rather than its catalytic activity, is required for Treg cell function; specifically, TCR activation causes ZAP-70 phosphorylation, which results in the activation of a series of proteins that recognize its phosphorylation sites, enhancing Treg/Tconv interaction, thus promoting Treg cell suppressive activity (31) (Fig. 1).

Another protein involved in TCR-dependent pathway and associated with both immunodeficiency and autoimmunity is represented by LCK, which is a Src family kinase pivotal for T cell activation and acquisition of effector functions. Recent findings have shown that complete deletion of *LCK* causes an immunodeficient phenotype, whereas partial attenuation of LCK function allows the maturation of Tconv cells but not regulatory T cells, leading to intestinal inflammation (32).

Together with LCK, also other TCR-associated molecules play a role in regulating tTreg cell generation: in particular, a knock-in mutation of LAT protein at Tyr136 leads to a lymphoproliferative syndrome with a severe deficit of Treg cells both in the thymus and in peripheral lymphoid organs (33). Similar results have been also observed in other experimental models, showing that LAT mutations (altering its interaction with PLC-γ1) inhibited Foxp3 expression together with Treg cell development and suppressive function, leading to severe autoimmune disease (33, 34). In line with this evidence, Fu *et al.* have shown that mice with a T cell–specific deletion of *PLC-γ1* were characterized by impaired generation and function of Treg cells and developed autoimmune disease; on the contrary, enhancement of diacylglycerol-mediated signaling, a second messenger produced by activated PLC-γ, has been shown to increase Treg cell suppressive activity (35, 36) (Fig. 1).

**Figure 2:**
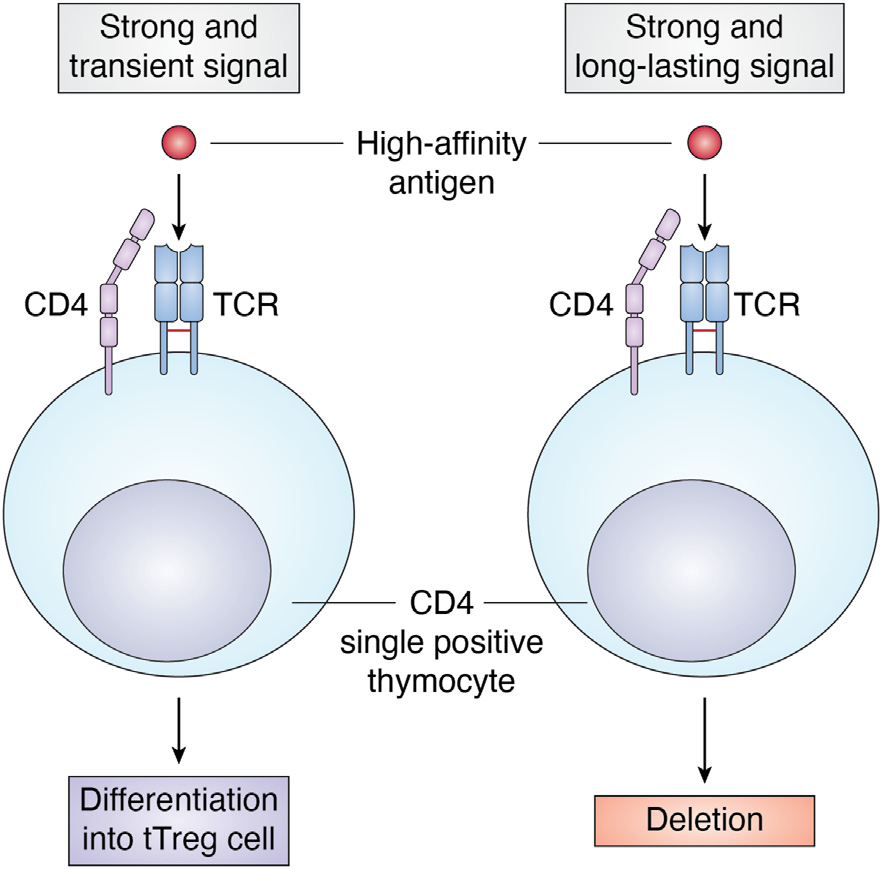
**TCR signaling and tTreg cell differentiation.** Thymic Treg cell differentiation from CD4 single-positive (SP) cells is sustained by a strong but transient TCR activation mediated by high affinity ligands, according to the “hit and run” model; in contrast, strong but long-lasting TCR-activating signals promote T cell clonal deletion. TCR, T cell receptor; Treg, regulatory T cell; tTreg, thymic-derived Treg cell.

Accordingly, signals that negatively regulate TCR-mediated pathways have opposite effects on Treg cell generation. Indeed, deletion of the tyrosine phosphatase Src homology region 2 domain–containing phosphatase-1 (*SHP-1*), a known negative regulator of TCR-mediated signaling, or exposure to increasing concentrations of stimulating peptide, resulted in an increased percentage of CD4^+^Foxp3^+^ cells in the thymus (37), and similar results were also found in mice bearing a deletion of CD5, another negative regulator of TCR signaling (38). Although the above data showed that attenuation of the TCR signaling strength correlates with decreased tTreg cell generation, it has recently been reported that mice with a mutant ζ chain (lacking functional ITAMs) displayed impaired negative selection of autoreactive thymocytes on the one hand and a significant increase in tTreg cell number on the other. At the molecular level, the described ζ chain mutation has been associated with reduced phosphorylation of ERK, Cbl, and protein kinase B or Akt, with little effects on calcium mobilization and activation of c-Jun N-terminal kinase, p38, and nuclear factor kappa-light-chain-enhancer of activated B cells, thus suggesting a selective and different role of ζ ITAMs on specific TCR-downstream pathways. In this context, the increased generation of Treg cells in these mutant mice could be related to specific attenuation of pathways able to inhibit Foxp3 expression, such as Akt (39). These data suggest that both inhibitory and activating molecules co-exist in TCR signaling pathway and participate in the control of tTreg cell generation with a different activation threshold. Specifically, the induction of tTreg cell is sustained by a strong but transient TCR activation mediated by high affinity ligands, according to the "hit and run" model (40, 41), which suggests that strong, but short-lasting TCR stimulation induces Foxp3-activating molecules and inhibits negative regulators of Foxp3 (such as Akt); in contrast, strong but long-lasting TCR-activating signals promote T cell clonal deletion (Fig. 2). Accordingly, thymic CD4^+^ T cells whose TCR is stimulated by poorly expressed and high affinity self-antigens, after transient activation, undergo a resting period in which they upregulate Foxp3 expression (40, 41).

**Figure 3:**
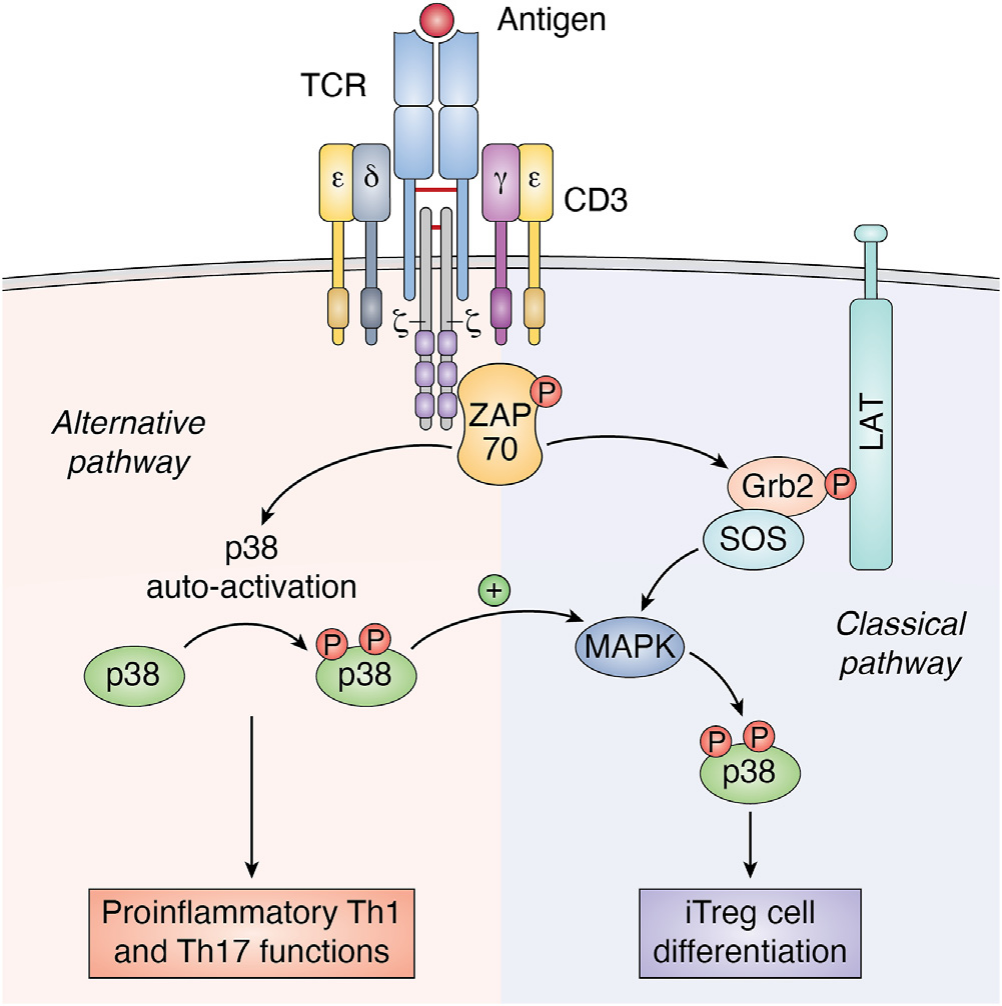
**The coexistence of the classical and alternative p38 activation pathways is necessary to maintain a balance between pro-inflammatory and anti-inflammatory responses.** TCR stimulation activates two distinct p38 signaling pathways: the ‘classical’ pathway involving ZAP-70 kinase, LAT signalosome assembly, SOS recruitment, and activation of MAPK cascades, and the ‘alternative’ pathway, in which p38 is directly activated by ZAP-70. Indeed, TCR-induced ZAP-70 activation mediates p38 phosphorylation at Tyr323, inducing its auto-activation. Activation of p38 through the ZAP-70–dependent alternative pathway lowers the activation threshold for the classical pathway, leading to full p38 activation. Inhibition of the classical p38 pathway by SOS deletion leads to a reduction in Treg cell differentiation, whereas the alternative pathway is required to maintain pro-inflammatory T helper functions, thus suggesting that both pathways work in concert to balance pro- and anti-inflammatory T cell responses. TCR, T cell receptor; Treg, regulatory T cell.

These data are consistent with the observations of Jun *et al.* reporting that TCR activation can induce differentiation of CD4^+^ T cells into proinflammatory or regulatory T cells, depending on the molecular mechanism by which p38 kinase is activated after antigenic TCR stimulation. Specifically, TCR-mediated activation of p38 has been shown to occur through phosphorylation mediated by “classical” kinases or through ZAP-70–dependent “alternative” mechanisms; perturbation of the classical or alternative pathways had opposite effects on CD4^+^ T cell differentiation. Indeed, attenuation of the classical pathway led to altered IL-2 production, reduced Treg cell differentiation, and exacerbation of experimental autoimmune encephalomyelitis whereas, the alternative pathway was preferentially required for T helper (Th) 1 and Th17 pro-inflammatory functions (42) (Fig. 3). Another mechanism by which high-affinity TCR signals can promote Treg cell development involves members of the tumor necrosis factor receptor superfamily. Indeed, developing thymocytes, characterized by TCRs with high affinity for self-ligands, exhibited increased expression of GITR, OX40, and tumor necrosis factor receptor-2, which enhanced their ability to compete for limited amounts of IL-2, thus providing a selective advantage for their differentiation into Treg cells (43).

### Role of TCR signaling in peripheral Treg cell induction

The intrathymic generation of Treg cells mediated by the above-described processes does not appear to be the only way in which these cells can be obtained, as suppressor T cells can also derive from the conversion of naive CD4^+^CD25^−^ T cells in the periphery. TCR-mediated signaling pathways also play a central role in regulating the induction and expansion of pTreg cell and are required to sustain the transcription of genes specifically expressed in activated Treg cells, such as interferon regulatory factor (*IRF4*) (44, 45). In addition, it has been shown that continuous TCR signals in the periphery are required to sustain Treg cell activation and suppressive function (46), but also weak TCR stimulation can promote their generation. Specifically, a low dose of a high-affinity TCR ligand represents the optimal quantitative and qualitative signal to induce a persistent population of Treg cells *in vivo*; moreover, also a chronic low-dose antigen exposure is capable of generating pTreg cells from naive T cells that are phenotypically and functionally similar to those generated in the thymus (47, 48).

Miskov-Zivanov *et al.* reported that there is an optimal “time window” of TCR stimulation for pTreg cell differentiation. The authors developed a logical network model of TCR signaling aimed at investigating the role of antigen dose and time of stimulation in determining T cell fate. Specifically, they found that restricting the time of TCR activation (achieved by antigen removal) resulted in three stable T cell fates (Th, Treg, and inactive) with relative proportions depending on the duration of stimulation (49). In the periphery, Foxp3 is induced more efficiently when T cells are stimulated under “sub-immunogenic” conditions (by low antigen doses with suboptimal dendritic cell (DC) activation), whereas stimulation with high doses of antigen results in increased T cell proliferation but reduced conversion into Foxp3^+^ cells, suggesting the presence of an inverse correlation between the number of cell divisions and the efficiency of Foxp3 induction (50). At the molecular level, this inverse correlation could be related to the cell cycle–dependent recruitment of DNA methyltransferase 1 and the subsequent CpG DNA methylation, resulting in the proliferation-dependent loss of Foxp3 expression (51). In support of this evidence, attenuation of TCR signaling strength by inhibition of ITK (a kinase involved in the TCR-induced pathway) has been shown to impair Th17 differentiation on one side, sustaining Treg cell differentiation on the other. ITK-deficient CD4^+^ T cells exhibited reduced TCR-induced phosphorylation of mTOR targets, such as Akt and ribosomal protein S6, resulting in reduced expression of hypoxia-inducible factor-1α and alterations in intracellular glycolytic metabolism (52) (Fig. 1). These data were consistent with previous experimental observations showing that inhibition of mTOR or Akt in CD4^+^ T cells promoted Foxp3 expression and Treg cell generation *via* multiple mechanisms (53, 54). The process by which TCR-mediated signaling strength promotes the induction of either Th or Treg cells involves the control of Akt activity. Specifically, depending on the strength of the TCR signal, Akt can interact with different substrates determining alternative cell fates (55). At the chromatin level, continuous TCR signaling and constitutive activation of the PI3K–Akt–mTOR pathway results in loss of the “permissive chromatin mark” for potential Foxp3 expression; in contrast, premature suspension of TCR signaling and inhibition of PI3K or mTOR pathways result in Foxp3 induction associated with permissive posttranslational histone modifications, such as di- and tri-methylation of histone H3 lysine 4 (H3K4me2 and -3) near the *Foxp3* transcription start site and within the 5′ UTR (56). Consistent with this finding, Turner *et al.* have shown that functionally suppressive Foxp3^+^ Treg cells can be both induced and expanded *in vitro* by DCs presenting low doses of antigen and thus inducing weak TCR signaling. In contrast, exposure to high doses of antigen resulted in expansion of Foxp3 cells, characterized by strong activation of the Akt–mTOR pathway (57). Advances in the understanding of TCR-mediated signal transduction pathways and how these signals are differently regulated in Treg cells *versus* other T cell subsets could be exploited to selectively target Treg cells, with the aim of developing novel therapeutic regimen for cancer and autoimmune diseases.

## CD28–CTLA-4 pathway involvement in the regulation of T cell activation

**Figure 4:**
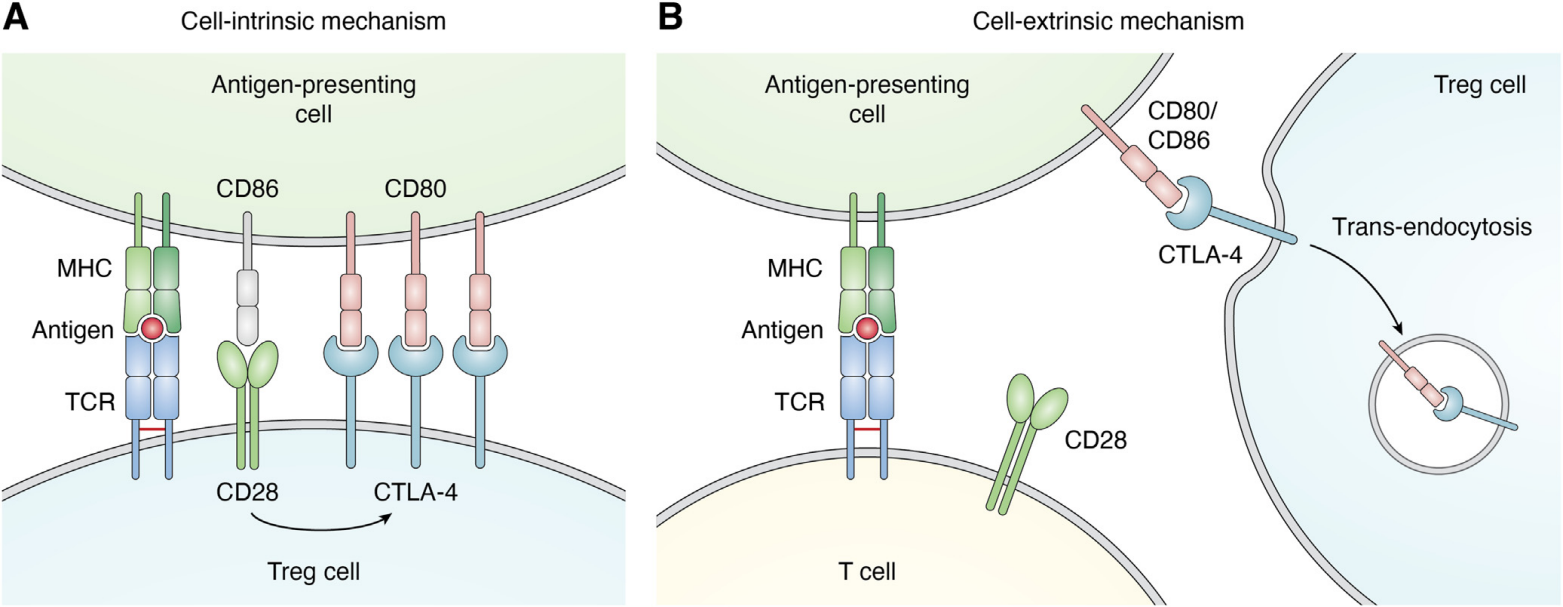
**Cell intrinsic and extrinsic regulation of T cell activation.***A*, high levels of CTLA-4 expression on Treg cell surface selectively inhibit the CD80–CD28 interaction as CTLA-4, by binding to CD80, prevents its binding to CD28, thus leaving the molecule CD86 as the only available ligand for CD28. In addition, the CD28/CD86 interaction further supports the suppressive phenotype of Treg cells by inducing the expression of suppressive markers (including CTLA-4 itself). *B*, Treg cells can inhibit T cell activation through a mechanism of CTLA-4–mediated trans-endocytosis of CD80 and/or CD86 from the APC surface, thereby preventing co-stimulation of CD28 on the T cell surface, thus inhibiting T cell activation. APC, antigen presenting cell; Treg, regulatory T cell.

As previously mentioned, regulation of TCR-mediated signals also involves co-stimulatory/co-inhibitory molecules, such as CD28 and CTLA-4, which balance protective immunity, regulating immunological self-tolerance, and tissue damage. CD28 is a 44 kDa type I transmembrane protein expressed on the surface of the majority of naïve CD4^+^ and CD8^+^ T cells and represents the major costimulatory molecule required, together with TCR engagement, for the generation of effector T cell responses and immunological memory. More specifically, CD28 stimulation, achieved through its interaction with CD80 and CD86 ligands expressed by APCs, triggers and coordinates a series of metabolic pathways sustaining the energetic demand for rapid cell divisions (58). Conversely, TCR engagement in the absence of co-stimulation results in functional inactivation of T cells upon antigen encounter, leading to a vital but unresponsive state known as T cell anergy, a mechanism that contributes to the maintenance of peripheral self-tolerance (59, 60). The level of CD28 co-stimulation plays a key role in T cell decision process following antigen encounter, as high levels of co-stimulation lead to T cell activation against potential pathogens, whereas a weak CD28 co-stimulation induces T cell anergy and peripheral tolerance to self-antigens. Regulation of CD28 pathway activation is strictly dependent on the expression of both CD28 and CTLA-4 on T cells and CD80, CD86, and MHC on APCs (61). CTLA-4 (CD152) is a type I transmembrane glycoprotein homologous to CD28, with whom it shares a conserved hexameric motif MYPPPY, part of the ligand-binding site (62). While CD28 is constitutively expressed by T cells, CTLA-4 expression is induced only upon activation and both molecules are able to interact with CD80 and CD86 ligands expressed on APCs, albeit with different affinity. Unlike CD28, the binding of CTLA-4 to its ligand generates an inhibitory signal that prevents T cell activation (63). The sharing of the same ligands suggests that the functions of CD28 and CTLA-4, although opposite, are closely interconnected and finely tuned to control T cell activation. The signals mediated by these molecules are controlled by the relative expression levels of CD28 and CTLA-4 on one hand and by the binding affinity for their shared ligands on the other, with CTLA-4 having a significant higher affinity for both CD80 and CD86 than CD28 (64). The interplay between CD28 co-stimulation and CTLA-4 immunosuppressive function plays a pivotal role in the control of autoimmune responses and the maintenance of immunological self-tolerance. CD28 and CTLA-4 cooperation is also required for proper thymic selection of natural Treg cells and for deletion of autoreactive T cells. Thymocyte negative selection occurs upon high-avidity engagement of the TCR by self MHC-peptide complexes, in the presence of additional signals such as CD28 co-stimulation (65, 66, 67, 68), although some papers have failed to highlight an indispensable role of CD28 in the negative selection process (69, 70). Furthermore, CD28-mediated pathway not only modulates the strength of TCR signaling but also coordinates distinct signals delivered to the TCR to drive Treg cell precursor maturation and prevent their clonal deletion (71, 72). CTLA-4, in turn, shapes the TCR repertoire of autoreactive T cells during thymic selection and ensures that T cells with high TCR avidity can be selected to become Treg cells (73). More specifically, CTLA-4, expressed by the corticomedullary region of the thymus, has been shown to attenuate the strength of TCR-mediated signals, counteracting the effects of CD28 and preventing the elimination of those clones that should be eliminated due to their high affinity for self-antigens. This process plays a crucial role in modulating the level of TCR avidity required for thymic selection and, consequently, for Tconv and Treg cell development (73). Indeed, although CD28 costimulatory pathway is required for optimal T cell activation and therefore has been implicated in the induction and progression of autoimmune diseases, it is also required for the generation and the homeostasis of Treg cells. Several studies performed in mice showed that CD28 inhibition led to a reduction in Treg cell number with important implications for allograft rejection (74). Moreover, CD28-deficient Treg cells expressed low levels of CTLA-4, PD-1, and C-C chemokine receptor type 6, displaying impaired suppressive function (75, 76). In parallel, Treg cells in CTLA-4–deficient mice were highly proliferative, consistently with a strong CD28 co-stimulation (not counterbalanced by the inhibitory activity of CTLA-4) although functionally impaired, thus leading to an uncontrolled T cell activation (77, 78, 79) (Fig. 1). Accordingly, CTLA-4–deficient mice developed a lethal autoimmune lymphoproliferative disorder, and different experimental approaches aimed at blocking CD28 pathway ameliorated their pathological phenotype (58, 80) (Fig. 1). All these data suggest that the establishment of central tolerance as well as the maintenance of peripheral tolerance are strictly dependent on both CD28 and CTLA-4 pathways and, even more, on their mutual counterbalancing interplay. Several molecular models have been proposed to describe the antagonistic role of CTLA-4 towards CD28 signaling. Specifically, CTLA-4 is able to hamper CD28 signaling also *via* a “cell-extrinsic” mechanism (Fig. 4) in which CTLA-4 can capture CD80 and CD86 ligands from the cell surface *via* trans-endocytosis with their consequent degradation, resulting in the inhibition of CD28 co-stimulation (81, 82). A “cell-intrinsic” mechanism of CTLA-4–mediated modulation of CD28 has been also described (Fig. 4) and is based on a direct competition for CD80 binding. High levels of CTLA-4 selectively impair CD80–CD28 interaction, due to the higher affinity of CD80 for CTLA-4 than CD28. Consequently, in cells expressing high levels of CTLA-4, such as Treg cells, CD80 is preferentially engaged by CTLA-4 and therefore CD86 becomes the main ligand available for CD28 co-stimulation, despite its lower affinity for this molecule. CD86-CD28 binding supports Treg cell proliferation and regulatory function by maintaining high expression of suppressive markers such as CTLA-4, ICOS, and OX40 (64). Consistently, CD80–CD28 interaction can be rescued in CTLA-4–deficient Treg cells, resulting in Treg cell expansion with impaired suppressive function (83, 84).Therefore, CD86 represents the preferential CD28 ligand selectively required for Treg proliferation, homeostasis, and activation.

However, the preference for CTLA-4 or CD28 binding and, consequently, the activation of downstream signaling is not only due to CD80 and CD86 levels but also to their different expression patterns on APCs. Indeed, CD86 is constitutively expressed on APC, whereas CD80 is mainly upregulated upon activation, and a differential cell-specific expression of these two molecules exists, with possible implications for preferential activation of either CD28 or CTLA-4 pathway (62, 63). In addition to the direct competition with CD28 for the shared ligands, CTLA-4 also interferes with the translocation of CD28 to the so called “immunological synapse” (IS), a specialized junction at the T cell–APC interface, crucial for the functional maturation and activation of T cells (85, 86). The IS consists of clusters of TCR-associated receptors and their downstream signaling molecules, in which TCR signals converge to initiate T cell responses. The IS includes a central supramolecular activation cluster (cSMAC), where the co-stimulatory signal originates and is finely regulated by competition between CD28 and CTLA-4. More specifically, CD28-mediated T cell co-stimulation occurs in a TCR-CD3 low-density region (CD3^lo^) of cSMAC, following specific recruitment of protein kinase C-q (PKCq). In this context, CTLA-4 negatively regulates T cell activation through a dynamic mechanism in which it translocates from the secretory granules to the IS upon TCR engagement, accumulates in the CD3^lo^ region of the cSMAC, and inhibits CD28 and PKCq recruitment. This mechanism has been observed both in Tconv and Treg cells, in which the constitutive expression of CTLA-4 results in the lack of PKCq translocation to the cSMAC, thus contributing to Treg cell *in vitro* anergic state (61, 86). Although several mechanisms have been proposed, how and whether CTLA-4 can exert its regulatory function at the molecular level remains still debated. However, it is now well accepted that CTLA-4 represents an essential checkpoint molecule for the control of autoimmunity, mainly because of its contribution to Treg cell generation and acquisition of suppressive function, which require a balanced integration of inhibitory and co-stimulatory signals coordinated by the CTLA-4–CD28 pathway.

In conclusion, given the relevance of CTLA-4–CD28 pathway in the regulation of TCR signaling, a precise understanding of its impact on immune system function may provide important insights on whether and how its modulation can suppress autoimmunity and promote anti-tumor immune response.

## Multifunctional roles for IL-2/IL-2R signaling in T cell tolerance

One of the main cytokines which supports TCR signaling in promoting Treg cell generation is IL-2, which was first identified in 1976 as a crucial T cell growth factor, exerting a wide range of actions, including regulation of cell survival, proliferation, differentiation, and acquisition of T cell effector functions (87, 88).

IL-2 is mainly produced by activated T cells after engagement of the TCR and the costimulatory molecule CD28 and mediates its biological functions through the binding to its high affinity receptor (IL-2R) which consists of three subunits: the α-chain (IL-2Rα, also known as CD25), the β-chain (IL-2Rβ, also known as CD122), whose expression is dependent by TCR-signaling, and the common cytokine receptor γ-chain (γc, also known as CD132), which is constitutively expressed by T cells (89, 90, 91). The IL-2/IL-2R complex activates downstream signaling pathways through the association of the tyrosine kinases JAK1 and JAK3 with the cytoplasmic tails of IL-2Rβ and γc, leading to JAK cross-phosphorylation and recruitment of members of the STATs family (92, 93, 94). IL-2 is able to activate several members of the STAT family, including STAT1, STAT3, and STAT5, which is the predominant IL-2 signaling–associated molecule (95). Upon recruitment, two STAT5 isoforms (STAT5A and STAT5B) are activated *via* JAK-mediated phosphorylation of the tyrosine 694 (STAT5A) and 699 (STAT5B) residues, which induces their dimerization and nuclear translocation, with consequent activation of their transcriptional activity, elicited through their binding to DNA or recruitment of other co-factors (96). In addition to tyrosine 694/699, a number of serine residues within STAT5A and STAT5B have been also identified as phosphorylation sites, including S127/128 (STAT5A), S725 (STAT5A), S730 (STAT5B), and S780 (STAT5A). While IL-2–dependent phosphorylation of these sites has been suggested to modulate STAT5 transcriptional activity, their precise functions and roles in T cell regulation are still under investigation (97, 98, 99). Although STAT5 is a critical downstream mediator of IL-2 signaling, constitutively active STAT5 is not sufficient *per se* to reproduce all the IL-2 biological effects, as IL-2–mediated pathways include other signaling cascades, such as Raf-ERK MAP kinases, PI3K-Akt, and mTOR (87, 97, 100, 101).

**Figure 5:**
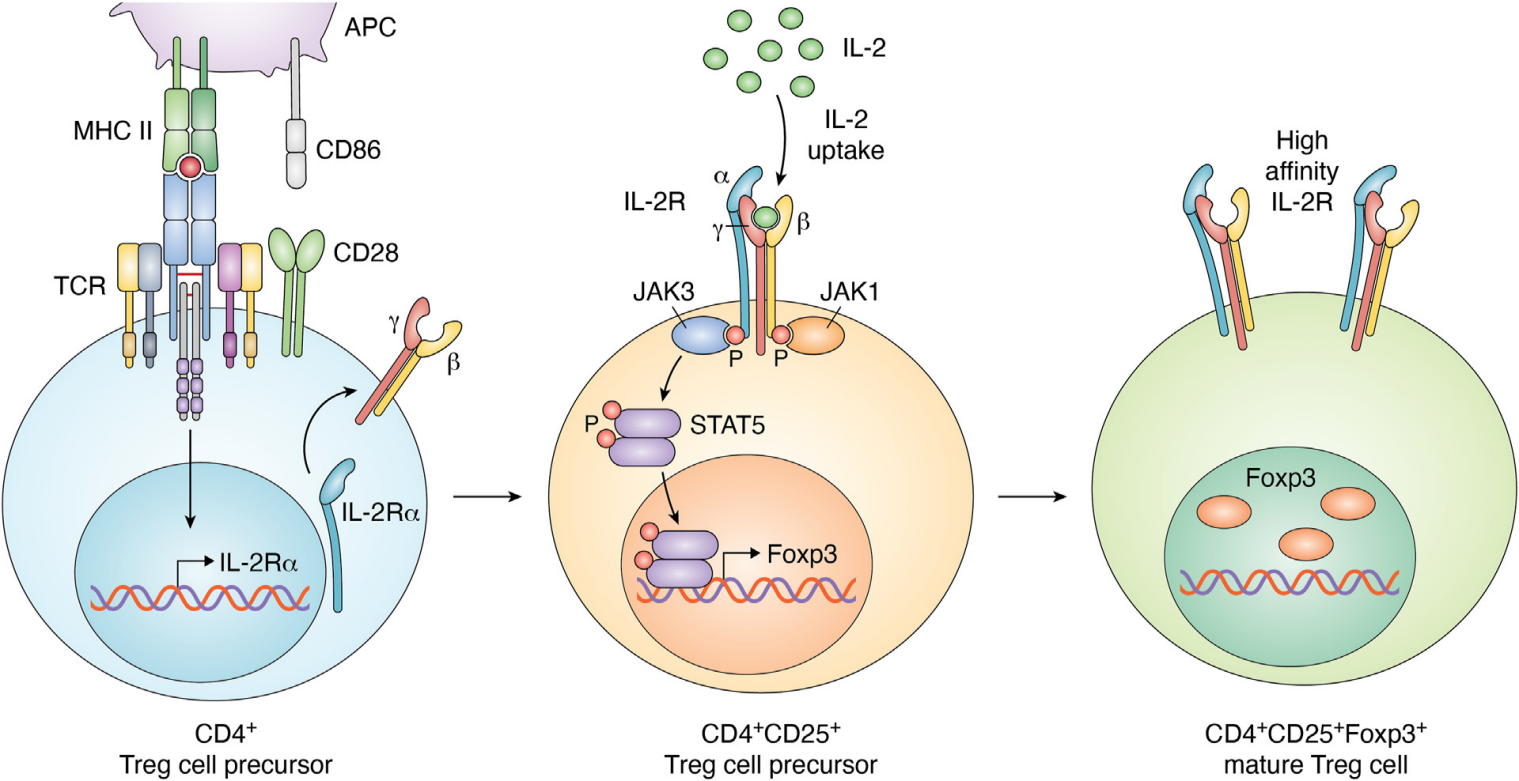
**Two-step model promoting Foxp3**^**+**^**Treg cell differentiation.** Thymic Treg cell development involves a two-step process: the first step is cytokine-independent and is induced by strong TCR stimulation occurring in developing CD4-single positive thymocytes (*blue cell*). This phenomenon leads to upregulation of α-chain of the high affinity IL-2 receptor (CD25), generating CD4^+^CD25^+^ Treg cell precursors (*orange* cell), which do not express Foxp3. The second cytokine-dependent step results in the upregulation of Foxp3, driving the conversion of Treg cell precursors into mature Treg cells (*green* cell). TCR, T cell receptor; Treg, regulatory T cell.

The dynamic ability of IL-2 to either promote or repress specific T helper cell programs depends on several mechanisms, including modulation of both cytokines and cytokine receptors expression, as well as negative regulation of cell type–specific transcription factors with activation of precise metabolic programs. In this regard, IL-2R–mediated signaling display a key role in the control of both effector T and Treg cell responses (102, 103, 104). Indeed, IL-2 contributes to optimal clonal expansion of activated T cells, driving their terminal differentiation and controlling T cell memory development and survival (91). IL-2 is also critical for Treg cell development, homeostasis, proliferation, and maintenance in the peripheral tissues, as it has been shown to sustain Foxp3 expression, the key transcription factor defining Treg cell identity and required for their function (105, 106, 107), despite contrasting data from Fontenot *et al.* have shown that IL-2 signaling is dispensable for the induction of Foxp3 expression in thymocytes (87, 106). All these IL-2–mediated effects on Treg cells are also due to their constitutive CD25 expression, which allows them to respond to low doses of IL-2 and sustains Treg cell function and CTLA-4 expression (91, 107). The process of tTreg cell generation is controlled by a tight network of relationship among TCR-mediated signal, IL-2R expression, and IL-2-mediated Foxp3 induction; specifically, during thymic Treg cell development, in a first cytokine-independent step, TCR- and CD28-mediated signaling generates IL-2–responsive Treg cell progenitors lacking Foxp3 expression but characterized by CD25 upregulation (Fig. 5). This high CD25 expression makes the precursors competitive for IL-2 uptake, which directly induces Foxp3 expression and finalizes Treg cell development. In the second step, in a TCR-independent but IL-2/STAT5–dependent process, IL2 mediates the rapid conversion of these Treg cell precursors into mature Foxp3^+^Treg cells (108, 109). Gomez-Rodriguez *et al.* suggested a possible mechanism by which IL-2 may regulate TCR signaling during Treg cell induction. Indeed, the authors showed that the IL-2–inducible T-cell kinase ITK mediated the cross-talk between TCR and IL-2 signaling, thereby influencing the balance between Th17 and Treg cells (52). Although Treg cells require TCR signaling to proliferate, in the presence of elevated IL-2 levels or when STAT5 is selectively activated in Treg cells, their division can occur independently of MHC class II and TCR signaling (110).

Several studies have shown that IL-2, together with transforming growth factor (TGF)-β, is an essential polarizing cytokine involved in inducible Treg cell differentiation, as its neutralization abrogated this process, even in the presence of TGF-β (111, 112, 113). Mechanistically, IL-2 has been shown to control the stability of Foxp3 expression in TGF-β–induced Treg cells *in vivo*, by promoting the demethylation of the conserved non-coding sequence 2 region (also known as Treg cell–specific demethylated region or TSDR) within the *Foxp3* gene (114). As we previously reported, STAT5 is considered a critical mediator of IL-2 signaling: specifically, its deficiency is associated with impaired Treg cell generation and its transient activation has proven to be sufficient to increase Treg cell number even in IL-2–deficient mice (115, 116). In addition, mice over-expressing a constitutively active form of STAT5B are protected from graft-*versus*-host disease and display increased Treg cell number with enhanced suppressive function, as compared to WT mice (117, 118). Supporting these observations, STAT5B knocking down in human primary T cells results in reduced Foxp3 expression; indeed a human rare autosomal recessive disease, characterized by STAT5B deficiency, has been associated with chronic lung disease, autoimmunity, and impaired Treg cell number and function (119) (Fig. 1).

The dysregulation of IL-2–mediated pathways has been identified as a key feature in the pathogenesis of several autoimmune disorders, resulting in a reduced number and altered function of Treg cells (Fig. 1). This concept is supported by observations showing that polymorphisms altering IL-2 signaling in humans are associated with autoimmune diseases, including type 1 diabetes, celiac disease, multiple sclerosis (MS), Graves’ disease, Sjögren’s syndrome, and rheumatoid arthritis (120, 121, 122, 123). Specifically, in subjects with type 1 diabetes, *IL-2RA* polymorphisms are associated with reduced soluble (s)IL-2R levels and lower STAT5A phosphorylation. Also, the diabetes-associated *IL-2RA* haplotype (rs12722495) correlates with reduced IL-2 responsiveness, impaired Foxp3 expression, and altered Treg cell suppressive function (124, 125). Overlapping results have been also obtained in MS, in which the rs2104286 polymorphism in the *IL-2RA* locus has been associated with a higher risk of developing the disease and with increased expression of IL-2Rα on naïve Th cells, which preferentially differentiate into encephalitogenic granulocyte-macrophage colony-stimulating factor–producing effector Th cells (121, 126).

Finally, IL-2 has been shown to play a critical role also in sensitizing T cells to antigen-induced cell death (AICD), one of the mechanisms responsible for the peripheral deletion of autoreactive T cells and induction of peripheral tolerance (127). Indeed, although IL-2 is an important T cell growth and survival factor, it can also limit the magnitude of T cell response, by inducing the upregulation of Fas, its ligand (FasL), and tumor necrosis factor receptor expression and concomitantly downregulating the cysteine aspartic enzyme inhibition factor (C-FLIP), thereby promoting AICD. In support of this evidence, KO mice for IL-2 or IL-2Rα are not sensitive to T cell AICD (127) (Fig. 1). In addition, IL-2 has been shown to promote the synthesis of interferon (IFN)-γ, which is a critical cytokine for AICD (128). Specifically, Refaeli *et al.* found that CD4^+^ T cells lacking IFN-γ or the STAT1 transcription factor were resistant to AICD, as IFN-γ was required for caspase-8 production, involved in cell death induction. On the contrary, forced expression of caspase-8 was able to restore T cell sensitivity to AICD in STAT1-deficient mice (129).

All these data support the notion that IL-2 regulates T cell differentiation upon antigen stimulation and represents a critical driver of T cell fate decision.

## Role of CD45 in the modulation of TCR signaling and immune tolerance

CD45, the leukocyte common antigen, is a transmembrane tyrosine phosphatase expressed on all nucleated hematopoietic cells. It plays a key role in controlling T lymphocyte activation, cytokine production, and the thymic development by regulating TCR-mediated signaling. In this context, CD45 can both positively and negatively regulate T cell activation; indeed, it is involved in the activation of the Src family kinase LCK but paradoxically, it has also been implicated in the suppression of the TCR-activated pathway, by dephosphorylating signaling motifs within the TCR complex (130, 131, 132). CD45 has been shown to be differentially required for the development and function of B and T lymphocytes; indeed, the development of B cells appeared normal in mice homozygous for the *CD45*-exon6 mutation, whereas thymocyte maturation was blocked at the transition stage from immature CD4^+^CD8^+^ to mature CD4^+^ or CD8^+^ cells (133).

This phosphatase can also act as a negative regulator of cytokine receptor signaling; in particular, targeted inhibition of CD45 has been shown to associate with enhanced cytokine and IFN receptor–mediated activation of JAKs and STAT proteins (134). Like many other protein tyrosine phosphatases, CD45 consists of an extracellular receptor-like region and an N-terminal region accounting for different isoform variants (135, 136). Indeed, multiple isoforms are generated by a tightly regulated alternative splicing mechanism, involving specific exons, in particular exons 4, 5, and 6 and resulting in differential inclusion of segments A, B, and C, respectively (137). The distinct isoforms differ in the size of their extracellular domains, and T cells can express the high or low molecular weight form of CD45 (CD45RA or CD45RO, respectively), depending on their activation state; indeed, differentiation of naive T cells, which express different larger isoforms (including RABC, RAB, RBC, and RB), into memory T cells is accompanied by exon exclusion for the production of the short isoform RO with concomitant downregulation of CD45RB expression. Basadonna *et al.* have shown that anti-CD45RB treatment can induce long-term engraftment of transplanted islets in diabetic mice, with reduced insulitis and preserved islet integrity (138, 139). Specifically, this treatment resulted in a shift in CD45 isoform expression on T cells from higher to lower molecular weight isoforms (CD45RO), together with a shift from CD45RB^Hi^ to CD45RB^Lo^. These phenomena were also associated with increased expression of Th2-type cytokines, such as IL-4 and IL-10, suggesting that the change in CD45 isoforms induced by mAb treatment can cause a shift in the functional repertoire of responding T cells, skewing the immune response towards allograft tolerance (140). In another paper by the same group, it has been demonstrated that the beneficial effect of anti-CD45RB treatment on allograft survival during transplantation can act through mechanisms involving CTLA-4 upregulation, as its inhibition reverted the phenomenon, thus demonstrating a direct link between CD45 and CTLA-4 (141).

In addition to the regulation of CTLA-4 expression, CD45 targeting can also control immune tolerance through the expansion of Treg cells; indeed, treatment with a tolerogenic anti-CD45RB mAb has been shown to increase homeostatic Treg cell proliferation in WT mice. Moreover, it has been also shown that CD45 ligation prolonged the interaction between Treg cells and DCs *in vivo*, reducing Treg cell motility and promoting nuclear translocation of nuclear factor of activated T cells, which is required for Treg cell proliferation. These data suggest that peripheral Treg homeostasis can be controlled *in vivo* through the specific targeting of CD45, which in turn promotes the interaction of Treg cells with DCs (142).

CD45 is also involved in the homeostatic control of T cell proliferation; in particular, van Vliet *et al.* have shown that the interaction between CD45 expressed by effector T cells with the C-type lectin receptor MGL expressed by APCs inhibits TCR-mediated signaling and cytokine response, resulting in decreased T cell proliferation and increased T cell death, thus preventing tissue damage and autoimmunity (143).

Recent experimental evidence has shown that a heterozygous C→G transversion at nucleotide 77 in exon 4/A of the gene encoding CD45 results in a defective CD45 alternative splicing, with altered expression of its isoforms, which has been associated with increased susceptibility to MS (144, 145) (Fig. 1). The same mutation has also been studied in the context of other autoimmune diseases; indeed, a significantly higher frequency of this mutation has been observed in patients with autoimmune hepatitis, characterized by the loss of tolerance against liver resident antigens (146). Furthermore, altered expression of the different isoforms of CD45 within CD4^+^ T cell subset has also been reported in children with infantile cholestasis and in patients with systemic lupus erythematosus (147, 148, 149).

In conclusion, while it is clear that CD45 can act as both a positive and negative regulator of T cell activation, several issues require further clarification, such as the functional role of the different CD45 isoforms and the regulation of their expression and alternative splicing. Understanding these mechanisms may be useful for the development of novel therapeutic tools able to modulate and control CD45 activity to direct T cell activation towards a pro-inflammatory or tolerogenic profile.

## Concluding remarks

TCR-mediated signaling plays a key role in the control of several mechanisms related to T cell biology, including their development, activation, differentiation, proliferation, and the acquisition of effector functions. Therefore, TCR-mediated pathways need to be finely-tuned to prevent the development of pathological conditions. In this context, positive and negative mechanisms of T cell activation are able to integrate signals from peptide:MHC complexes and cell surface accessory molecules, translating them into intracellular events that influence cell fate decisions. In the past few years, compelling experimental evidence has demonstrated that TCR signal strength can regulate CD4^+^ T cell differentiation also controlling the phenotype and effector functions of Th1/Th17 cells or Treg cells. In determining the precise differentiative cell fate, the activity of TCR signaling can control T cell activation or tolerance induction toward a particular antigen. In this context, both T cell hypo- and hyper-responsiveness, caused by abnormalities in TCR-signaling pathways, can lead to the development of autoimmunity. In light of all these considerations, mechanisms or molecules able to modify TCR-mediated signaling and its regulatory pathways might represent a potential therapeutic target to control T cell activation and possibly modulate their fate to drive the immune response towards an inflammatory or tolerogenic phenotype. In conclusion, further studies are needed to fully understand these regulatory mechanisms, which could allow us to modulate immune responses for the treatment of immune-mediated diseases, such as immunodeficiency or autoimmunity.

## Conflict of interest

The authors declare that they have no conflicts of interests with the contents of this article.
